# Odontogenic tumors: A Retrospective Study in Egyptian population using WHO 2017 classification

**DOI:** 10.4317/medoral.24661

**Published:** 2022-04-14

**Authors:** Leena Al-aroomy, Mona Wali, Mohamed Alwadeai, Eman El Desouky, Hatem Amer

**Affiliations:** 1 MSc, PhD, Oral and Maxillofacial Pathology, Faculty of Dentistry, Cairo University, Egypt; 2 Professor and head of Oral and Maxillofacial Pathology, Faculty of Dentistry, Cairo University, Egypt; 3 PhD candidate in Oral and Maxillofacial Surgery, Faculty of Dentistry Cairo University, Egypt; 4 Assistant lecturer in Oral and Maxillofacial Surgery, Faculty of Dentistry, Ibb University, Ibb, Yemen; 5 Lecturer of epidemiology and biostatistics, National Cancer Institute, Cairo University, Egypt; 6 Lecturer of Oral and Maxillofacial Pathology, Faculty of Dentistry, Cairo University, Egypt

## Abstract

**Background:**

Odontogenic tumors (OTs) are considered important among oral lesions because of their clinicopathological heterogeneity and variable biological behavior. The purpose of this retrospective cross-sectional study was to evaluate the frequency and distribution of different types of odontogenic tumors based on the current 2017 WHO Classification of Head and Neck Tumors over a period of 5 years. This was achieved by reviewing the records of Cairo's educational hospitals and institutions and comparing the results with findings in the literature.

**Material and Methods:**

The records of patients diagnosed with odontogenic tumors were obtained from six educational hospitals and a single institute in Cairo which included: Oral and Maxillofacial Pathology Department, Faculty of Dentistry, Cairo University; General Pathology Department, Faculty of Medicine, Cairo University; Oral Pathology Department, Faculty of Dentistry, Ain Shams University; Eldemerdash Hospital, Ain Shams University; El-Sayed Galal Hospital, Al-Azhar University; Ahmed Maher Teaching Hospital and National Cancer Institute. These records were reviewed over a 5-year (2014-2018) period and the odontogenic tumors were investigated for frequency, age, gender and site. The data were recorded, then analyzed using SPSS software.

**Results:**

Intraosseous (central) odontogenic tumors constituted 2.56% of all 8974 registered oral and maxillofacial biopsies. A total of 230 cases of OTs were collected and reviewed. Of these, 97.8% were benign and 2.17% were malignant. The mandible was the most commonly affected anatomic location. Ameloblastoma, with a predilection for the posterior mandible, was the most frequent odontogenic tumor (55.65%), followed by cemento-ossifying fibroma (14.78%) and odontoma (9.13%). Females were more commonly affected than males. Most of the patients were in the third and fourth decades of life. There were no peripheral odontogenic tumors diagnosed in this period.

**Conclusions:**

Some similarities and differences between our findings and those of previous studies of various populations were witnessed. OTs may greatly diverge according to the version of the classification used and by the sample size of the study. Retrospective analysis of the relative frequency of OTs in different countries will be helpful in enhancing the understanding of OTs, which is important for both oral maxillofacial surgeons and pathologists.

** Key words:**Odontogenic tumors, epidemiology, world health organization classification, oral pathology.

## Introduction

Odontogenic tumors (OTs) comprise a group of heterogeneous lesions with different histopathological characteristics and clinical manifestations. From a biological point of view, some of these lesions represent hamartomas with varying degrees of differentiation, while the rest are benign or malignant neoplasms with variable aggressiveness and potential to develop metastasis. Odontogenic tumors are lesions of the mandible and maxilla that must be considered as a part of the differential diagnosis of lesions that occur in the jaws (1-3).

The knowledge of the histological features of the different odontogenic tumors, as well as of their clinicopathological features recorded in diverse populations worldwide, are important points that may help to identify the groups at risk and possible factors associated with the development of these infrequent, but biologically complicated lesions (4). The first internationally accepted classification system for OTs was published in 1971 by the World Health Organization (WHO), which was reviewed and updated in 1992 and in 2005 (5). WHO classification of head and neck tumors (fourth edition) was the last update and was announced in early 2017 (6).

It is very important to form a set of criteria such as sex, age, and location of lesion for the management of OTs (7). OTs vary worldwide in incidence and provide country differences. In North America, South America and Europe, the frequency of odontogenic tumors was less than 3%, whereas in Africa and Asia, OTs constituted 9.6% and 8.99%, respectively (3).

To the best of our knowledge, there is a lack of studies in the English-language literature that describe the frequency of OTs in different parts of Egypt. For this reason, this study aimed to examine the prevalence of different types of odontogenic tumors in Cairo governorate, based on the updated WHO classification of Head and Neck Tumors (2017).

## Material and Methods

This retrospective cross-sectional study assessed the prevalence of OTs based on the WHO classification of Head and Neck Tumors (2017) (6). A 5-year (2014-2018) retrospective review of OTs was carried out. Information from case files and histopathology records of patients with OTs were retrieved from six educational hospitals and a single institute in Cairo, as follows: Oral and Maxillofacial Pathology Department, Faculty of Dentistry, Cairo University; General Pathology Department, Faculty of Medicine, Cairo University; Oral Pathology Department, Faculty of Dentistry, Ain Shams University; General Pathology Department, Faculty of Medicine (Eldemerdash Hospital), Ain Shams University; El-Sayed Galal Hospital, Al-Azhar University; Ahmed Maher Teaching Hospital and National Cancer Institute. These odontogenic tumors were assessed for age, gender and site. All cases of OTs were re-examined by the two pathologists (HA and LA) and the final diagnosis was modified according to the WHO classification of Head and Neck Tumors (2017). Descriptive statistical analysis was performed with all collected data using SPSS software (version 24; SPSS, Inc, Chicago, IL). Percentage and frequency Tables were used to describe the pattern of distribution and the allocation of different OTs according to different age groups, sex and sites.

## Results

From a total of 8974 oral and maxillofacial (OMF) biopsies registered during the 5-year period from January 2014 to December 2018, with the exclusion of incomplete medical records and records missing histopathological reports, only 230 cases (2.56%) were odontogenic tumors. Of the 230 cases, all the reported odontogenic tumors were centrally located. Besides, the majority of odontogenic tumors were benign accounting for 225 (97.8%), while the remaining 5 (2.17%) were malignant tumors.

Ameloblastomas (conventional “solid” and unicystic ameloblastoma) were the most frequent type of benign odontogenic tumors, accounting for 128(55.65%) followed by cemento-ossifying fibroma, odontoma, odontogenic myxoma, ameloblastic fibroma, adenomatoid odontogenic tumor, calcifying epithelial odontogenic tumor and odontogenic fibroma. Both ameloblastic fibrodontoma and cementoblastoma were the least commonly diagnosed cases. On the other hand, ameloblastic carcinoma and ameloblastic fibrosarcoma were the only two types of malignant OTs diagnosed (Table 1).

**Table 1:**
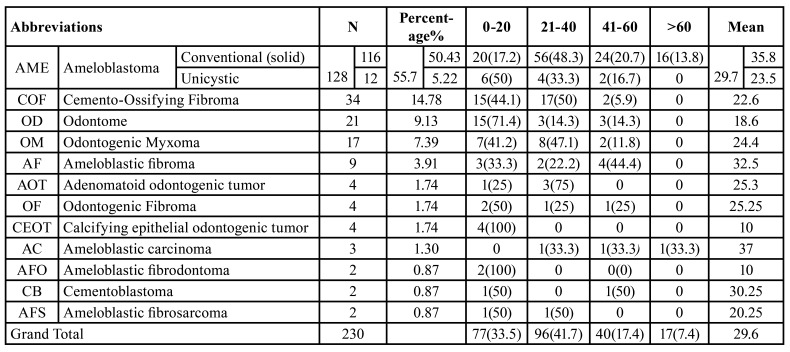
Frequency and age distribution of OTs in the present series.

The most common histological subtypes of conventional ameloblastoma were follicular accounting for 76(65.5%), followed by plexiform accounting for 23(19.8%). Acanthomatous and granular subtypes of conventional ameloblastoma were less commonly seen, constituting 12(10.3%) and 5(4.35%) respectively. Regarding the variants of unicystic ameloblastoma, the mural type was the most common, accounting for 6 (50%) followed by intraluminal type accounting for 4 (33.33%), while the luminal type was the least common, accounting for 2 (16.66%). Odontoma has two types, complex odontoma accounting for 12 (57.14%) and compound odontoma accounting for 9 (42.85%).

The age of patients ranged from 9 to 82 years with a mean age of 29.6 years. OTs were most often observed among patients in the second to fourth decades of life (96 cases; 41.7%) and least among elderly patients (17 cases; 7.4%) with ages within the 6th decade and above. The specific age ranges for each OT are shown in Table 1.

From the 230 OT cases, 119 (51.7%) occurred in females, while 111 (48.3%) were male patients. Cemento-ossifying fibroma, odontoma, odontogenic myxoma and ameloblastic fibroma were more frequently diagnosed among females. Ameloblastoma, odontogenic fibroma, ameloblastic fibrodontoma and ameloblastic carcinoma were observed more frequently among male patients. On the other hand, calcifying epithelial odontogenic tumor, adenomatoid odontogenic tumor, cementoblastoma and ameloblastic fibrosarcoma had equal distribution among both sexes (Table 2).

**Table 2:**
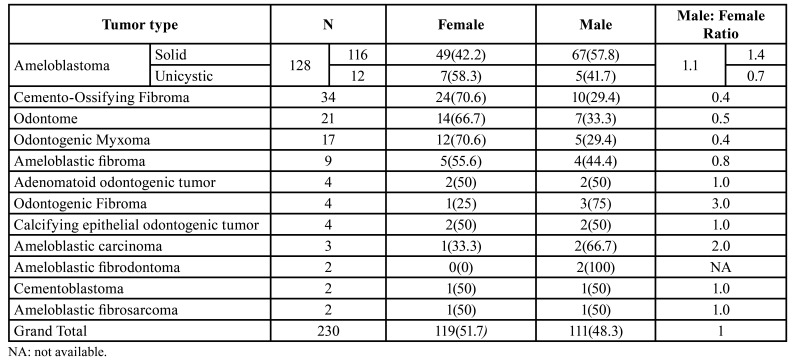
Distribution of OTs according to gender.

The mandible (177 cases; 77%) was more commonly affected than the maxilla (53 cases; 23%), with a mandible to maxilla ratio of 3.3:1. The posterior mandible was the most affected site, followed by the posterior maxilla, anterior maxilla and anterior mandible. Conventional and unicystic ameloblastoma, cemento-ossifying fibroma, odontoma, odontogenic myxoma, calcifying epithelial odontogenic tumor, ameloblastic fibroma, and ameloblastic carcinoma cases were most often seen in the mandible. Cementoblastoma and ameloblastic fibrosarcoma were exclusively seen in the posterior mandible, while adenomatoid odontogenic tumor cases were most commonly seen in the anterior maxilla. Odontogenic fibroma and ameloblastic fibrodontoma equally affected both jaws (Table 3).

**Table 3:**
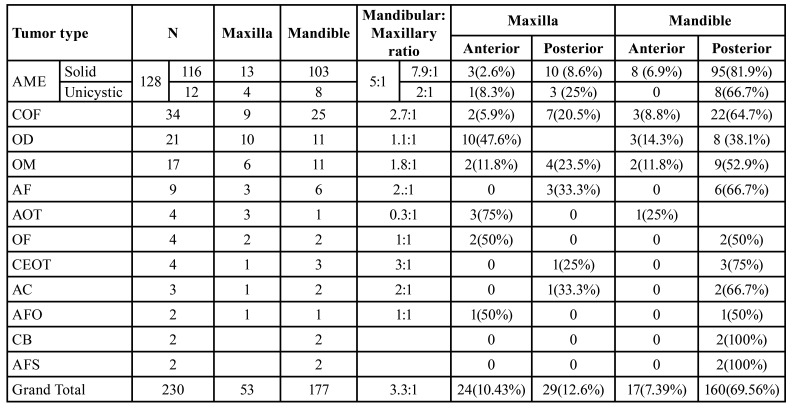
Mandibular to maxillary ratio and distribution of anterior and posterior regions of OTs.

## Discussion

Knowledge of the population-based prevalence and salient clinical features of odontogenic tumors is essential for accurate differential diagnosis and determination of related risk factors (1,5). The variation in the prevalence of oral lesions reﬂects differences in cultural and socioeconomic patterns between different countries that can inﬂuence the habits and diseases of a population. However, another factor that could explain the different prevalence of oral lesions is the fact that few studies describe the national proﬁle or the proﬁle of representative regions of a country and others report prevalence limited to a single dental or medical school, nursing homes or to institutionalized patients (8).

The average proportion of OTs among oral and maxillofacial lesions evaluated by histopathology increased from 3% (±2.9%) in studies that used the 1992 WHO classification of tumors to 4.0% (±1.3%) in those with the later classification (9). The mainstream of articles which were published after 2005 have used the 2005 WHO classification (7-14) except for few articles which used the latest 2017 classification (2,15,16). Therefore, in this study, we aimed to investigate the prevalence of OTs according to the WHO classification in 2017.

The relative frequency of odontogenic tumors in the present study was 2.56% of all OMF specimens recorded between January 2014 to December 2018. This relative frequency is similar to reports from studies conducted in Turkey (2.74%) (5) and India (2.17%) (11). However, another study conducted in Nigeria (17) showed a higher frequency of OTs (9.6%). Lower rates of OTs were observed in Europe (0.84%), North America (1.2 %) and South America (1.29%) (9).

These variations in the frequency of OTs may be the result of 2 main reasons: (1) higher number of reactive and inflammatory lesions are subjected to microscopic examination in developed countries, whereas only patients with incapacitating symptoms presented to the hospitals in developing countries (18); and (2) the changes that have occurred over the years in the WHO classification of OTs, which explain that some of the pathologic entities have been excluded, while new ones have been introduced.

In this study, the majority of OTs were benign, accounting for 225 (97.8%), which is in accordance with reports by Mosqueda-Taylor *et al*. (1), Sekerci *et al*. (5), Kebede *et al*. (12) and Gotur *et al*. (13) while the remaining 5 (2.17%) were malignant. The percentage of malignancy was lower than previous studies from Brazil (5.5%) (4), Ethiopia (19.6%) (12), and China (6.1%) (19).The observed differences in the distribution of malignant OTs might be due to the geographical and cultural variation among the different study populations (12).

The average age of OTs was found in the second to the fourth decades of life (29.6 years), similar to studies from Brazil (9), India (11), and Italy (20). Furthermore, approximately 84.2% of odontogenic tumors were observed during the 2nd to 4th decade of life, whereas only 3% cases were seen below 10 years of age. The reason may be due to the fact that most odontogenic tumors are commonly associated with the permanent teeth. However, studies conducted in Argentina (21) and Libya (22) reported significant involvement of children and adolescents. Overall, a higher tendency toward females (51.7%) was noticed in this study, corroborating the studies by Ochsenius *et al*. (23) and da Silva *et al*. (24). However, Kebede *et al*. (12) and Aregbesola *et al*. (15) reported male predilection.

In this study, the most affected site by the OTs was the mandible, with the mandibular to maxillary ratio of 3.3:1; these results were in accordance with other reports by Mascitti *et al*. (2), Silva *et al*. (8), Lima-Verde-Osterne *et al*. (9), Nalabolu *et al*. (11), Aregbesola *et al*. (15) and Rubini *et al*. (20). Furthermore, in our study, the posterior mandible was the most affected site (69.56%). These data were consistent with those of da Silva *et al*. (24) and Deepthi *et al*. (25) followed by posterior maxilla as second commonly affected site (10.43%).

Ameloblastoma (55.7%) was the most common type of OT observed, followed by cemento-ossifying fibroma (14.78%), odontoma (9.13%), and odontogenic myxoma (7.39%). This study revealed the highest incidence of ameloblastoma with 128 cases (55.7%), similar to reports by Sekerci *et al*. (5), Nalabolu *et al*. (11), Kebede *et al*. (12), Lawal *et al*. (14) and Daley *et al*. (26). The incidence of ameloblastoma in this study strengthens the belief that these lesions are more common in Africans and Asians than in Caucasians. This finding is in contrast to studies from Brazil, Italy and Chile (9,20,23). Regarding gender, our results showed that males were more commonly affected than females (M=72, F=56), in accordance to meta-analysis studies done in Africa (M = 650, F = 542), North America (M = 180, F = 124), and Asia (M = 2,218, F = 1915). Australia also reported a male predominance, but the difference was not statistically significant (M = 26, F = 15). Nonetheless, some authors have reported a female predilection in South America (M = 269/F = 307) and Europe (M = 84/F = 105) (27).

Furthermore, ameloblastoma was observed in all age groups with the peak incidence in the third decade, which is similar to other studies by Lima-Verde-Osterne *et al*. (9), Aregbesola *et al*. (15) and Jing *et al*. (28). A systematic review by Hendra *et al*. (27) documented that the mean age of ameloblastoma was in the third decade. In Europe (26.2%) and North America (34.0%), ameloblastoma mostly occurred at an older age (the fifth and sixth decades) while in Africa (32.8%), South America (29.7%) and in Asia, peak incidence was between the third and sixth decades (27). The age variation in ameloblastoma among countries may be due to the accelerated aging process in developing countries owing to poor nutrition and health care (10). Moreover, the posterior mandible was the most affected site, accounting for 74.3% of the cases. These results conform with other results found in the published literature (18,29).

Surprisingly, cemento-ossifying fibroma was the second most prevalent OT in our study accounting for 14.78%. This finding contrasts with reports from Italy (2), Brazil (9) and India (11).The peak incidence of age occurred between the second and fourth decades, with mean age of 22.6 years, which is similar to a study in India (16). Females were significantly more affected than males (M: F ratio of 0.4:1), as reported by Ahire *et al*. (16). Regarding the site of onset, cemento-ossifying fibroma occurred more frequently in the mandible with mandible to maxilla ratio of 2.7:1, which is in accordance with the study held in Italy (2), but opposite to another study from India (16).

Odontomas were recorded as the third most common OT in this study with a frequency of 9.13%, similar to what has been found in previous studies (5,9,11). However, studies conducted in Mexico (1), Chile (23) and North California (29) reported higher frequency of odontomas, reporting these lesions as the most prevalent OT in their studies (representing 34.6%, 44.7% and 75.9% respectively).

Overall, epidemiological data in the literature showed significant differences among countries. Ameloblastoma seems more common in Asian and African countries, while in North America, the most frequently diagnosed OT was odontoma (12). A reason for this discrepancy is the source of data. In Asian and African countries, odontogenic lesions are diagnosed and treated in maxillo-facial units, while patients from Europe and North America can be treated both in hospitals and dental schools (12,30). In particular, odontomas are commonly diagnosed on the basis of clinical and radiographic exams, without histological assessment, resulting in an underestimation of their frequency (12). The mean age of the odontomas was 18.6 years, which agreed with studies that showed that odontoma mostly occurs in younger individuals (29,30). Regarding gender, we observed that odontomas had a female predilection (66.7%), which is different from studies by da-Costa *et al*. (4), Ahire *et al*. (16) and Rubini *et al*. (20), but similar to the studies by Sekerci *et al*. (5) and Lima-Verde-Osterne *et al*. (9). In relation to the site, the mandible showed a slight predilection, with the mandibular to maxillary ratio being (1.1:1). These results are in agreement with those reported by Kebede *et al*. (12) and Rubini *et al*. (20); however, other authors reported a significant maxillary predilection (4,9). One study reported by Nalabolu *et al*. (11) found no differences between the mandible and maxilla.

The frequency of odontogenic myxoma in this study was 7.39%, which is in accordance with several studies from Mexico (1), Egypt (10) and Nigeria (17), which documented frequencies ranging from 6.5 to 17.7%. On the other hand, frequency of odontogenic myxoma in Brazil (4) and North California (29) was in the range of 2.2-4.9%. The mean age was 24.4 years, which is consistent with documented reports from previous studies (13,17). The preferred site for odontogenic myxoma was the mandible, with mandibular to maxillary ratio of 1.8:1. This result agrees with studies from India (13), Nigeria (17) and Northern California (29). However, Lima-Verde-Osterne *et al*. (9) found a maxillary predilection. The female gender predilection observed for odontogenic myxoma agrees with reports from previous Brazilian, Indian and Nigerian studies (4,13,17).

On the other hand, in this study, some odontogenic tumors demonstrated a much lower incidence rate, such as ameloblastic fibroma (3.91%), odontogenic fibroma (1.74%), calcifying epithelial odontogenic tumor (1.74%), adenomatoid odontogenic tumor (1.74%), cementoblastoma (0.87%) and ameloblastic fibro-odontoma (0.87%).

Regarding malignant odontogenic tumors, their frequency was significantly lower, accounting for 2.17%. Interestingly, Kebede *et al*. reported a significantly higher frequency of malignant OTs (19.6%). These authors hypothesized that the use of traditional medicines by some of these patients could have significantly delayed surgical treatments at late stages of disease. This diagnostic and therapeutic delay might have increased the risk of transforming benign OTs into malignant OTs (12).

We reported a low frequency of ameloblastic carcinoma (1.3%), similar to reports from Italy (1.1%) (20) and China (1.6%) (28), but in contrast with reports from Brazil (3.5%) (4) and Nigeria (2.2%) (17). Our results showed that the mean age for ameloblastic carcinoma was 37 years, which is much higher than that reported by Aregbesola *et al*. (15) and Rubini *et al*. (20). The male predilection observed for ameloblastic carcinoma agrees with reports from previous studies (4,20,25), but contrasts with studies conducted by Sekerci *et al*. (5) and Aregbesola *et al*. (15). The results of the current study aligned with several studies (4,15,25) in which the mandible was the most common site of involvement as opposed to Sekerci *et al*. (5) and Rubini *et al*. (20) in which maxilla was mostly affected. Furthermore, ameloblastic fibrosarcoma had a lower incidence (0.87%) with no gender predominance, the mandible was most frequently affected, and a mean age of 20.25 years was noticed, which is similar to a study by Aregbesola *et al*. (15).

## Conclusions

This analysis revealed some similarities and variances between our findings and those of previous studies of populations in Africa, Asia, Europe and the Americas. Although OTs may greatly differ in attribution to the version of classification used, they are also influenced by the study sample size. OTs differ according to genetic and/or environmental (epigenetic) factors. So, it was concluded that the knowledge of the relative incidence of odontogenic tumors in various parts of the world improves the understanding of the lesions, which contribute significantly in enhancing the concepts of treatment and prognosis.
